# Corneal Epithelial Remodeling after LASIK Measured by Fourier-Domain Optical Coherence Tomography

**DOI:** 10.1155/2015/860313

**Published:** 2015-04-28

**Authors:** Maolong Tang, Yan Li, David Huang

**Affiliations:** Center for Ophthalmic Optics & Lasers, Casey Eye Institute and Department of Ophthalmology, Oregon Health & Science University, Portland, OR 97239, USA

## Abstract

*Purpose*. To quantify corneal epithelial thickness changes after myopic LASIK by OCT.* Methods.* Epithelial thickness before and after myopic LASIK were measured by a Fourier-domain OCT system. Average central (within 1 mm diameter) and paracentral epithelial thickness (5~6 mm diameter) before and after LASIK were compared. Correlation between central epithelial thickness change and laser spherical equivalent setting was evaluated. An epithelial smoothing constant was estimated based on a mathematical model published previously.* Results.* Nineteen eyes from 11 subjects were included in the study. Eyes had myopic LASIK ranging from −1.69 D to −6.75 D spherical equivalent. The average central epithelial thickness was 52.6 ± 4.1 *μ*m before LASIK and 56.2 ± 4.3 *μ*m 3 months after LASIK (*p* = 0.002). The average paracentral epithelial thickness was 51.6 ± 6.6 *μ*m before LASIK and 54.8 ± 4.3 *μ*m 3 months after LASIK (*p* = 0.007). The change in average central epithelial thickness was correlated with laser spherical equivalent (*R* ^2^ = 0.40, *p* = 0.028). The epithelial smoothing constant was estimated to be 0.46 mm.* Conclusions*. Corneal epithelial thickens centrally and paracentrally after myopic LASIK. The extent of epithelial remodeling correlated with the amount of LASIK correction and could be predicted by a mathematical model.

## 1. Introduction

Corneal epithelium is able to alter its thickness to mask subepithelial stromal irregularities and maintain a smooth anterior surface of the eye. Laser refractive surgery such as LASIK alters the anterior corneal contour and leads to remodeling of corneal epithelium. We estimated the extent of the epithelial remodeling after LASIK with a mathematical model in a previous study [1]. However, in that study, we did not have the capability to measure the corneal epithelial thickness to validate the model directly. Instead, we constructed the model constants based on regression in manifest refraction after LASIK. Direct measurement of epithelial thickness change may help to better understand corneal epithelial remodeling after LASIK and improve LASIK ablation patterns with less regression and surgery-induced aberrations.

Previous studies used confocal microscopy to measure epithelial thickness, but the number of points measured was limited [2] and the measurement was time consuming [3]. Very high-frequency digital ultrasound was also used to map corneal epithelium and stromal thickness [4, 5]. However, because ultrasound cannot pass through air, this technique required immersing the cornea in a fluid bath. Though both confocal microscopy and very high-frequency digital ultrasound are feasible in measuring corneal epithelial thickness, they are not often used in routine LASIK because they required touching the cornea.

Optical coherence tomography (OCT) is a noncontact imaging technique based on principles of low-coherence interferometry. Its high axial resolution allows precise delineation of the different layers of the cornea. The current generation of OCT is based on Fourier-domain technique [6, 7]. In a recent article, we demonstrated that a commercial Fourier-domain OCT could automatically map the corneal epithelial thickness with good repeatability [8]. In this study, we use this algorithm to map corneal epithelial thickness for eyes before and after myopic LASIK surgeries. The changes in epithelial thickness were used to validate the smoothing model we mentioned above [1].

## 2. Materials and Methods

This prospective observational study was conducted at Doheny Eye Institute, Los Angeles, CA. The LASIK subjects enrolled in this study had no history of eye surgery and were comprehensively examined to exclude any eye diseases including dry eye. Soft contact lenses wearers were asked to stop wearing contact lenses at least two weeks prior to LASIK. The Institution Review Board of the University of Southern California approved the study. Informed consents were obtained from all subjects. The treatment of study subjects was in accordance with the tenets of the Declaration of Helsinki.

All subjects underwent uncomplicated LASIK for myopia and/or astigmatism. The laser settings were based on manifest and cycloplegic refractions calculated at the corneal plane. The same surgeon (David Huang) performed all LASIK procedures. The LASIK flap was created with a 60 kHz femtosecond laser (Intralase, Abbott Medical Optics, Santa Ana, CA). The femtosecond flap thickness was programmed to 110 *µ*m with a diameter of 9.0 mm and a 70-degree angled side cut. All flaps had a superior hinge. The stromal ablations were performed with VISX Star S4 IR CustomVue excimer laser (Abbott Medical Optics, Santa Ana, CA). The optical zone was set to 6.5 mm in diameter centered on the pupil center with blend/transition zones up to 8.0 mm.

To measure epithelial thickness before and after LASIK, a commercial Fourier-domain OCT system (RTVue, Optovue Inc., Fremont, CA) with a speed of 26,000 axial scans per second was used. It had axial resolution of 5 microns in tissue. A “Pachymetry + Cpwr” scan pattern (6 mm scan diameter, 8 radials, 1024 axial-scans each, repeated 5 times) centered at the pupil center was used to map the cornea. The entire scan pattern was completed in 1.58 seconds. The pupil was not dilated before OCT scans. During the scanning, the room lights were on. The subjects were asked to look straight ahead and fixate on the internal fixation target of the OCT system. The OCT scan pattern was repeated 2 times on each eye during the same visit. OCT scans were performed before LASIK and 3 months after. Corneal epithelial thickness on the OCT images was measured by an automated algorithm. The algorithm was described in a previous article [8] and was available in the commercial RTVue software. The average epithelial thickness over the central 1 mm diameter, 1~2 mm, 2~3 mm, 3~4 mm, 4~5 mm, and 5~6 mm annular zones, was used in the analysis.

Correlation of LASIK-induced change in epithelial thickness with the amount of spherical equivalent of LASIK correction was investigated. A smoothing constant [1] was estimated based on the slope of the correlation. Based on our epithelial smoothing model [1], the change in epithelial thickness could be calculated by applying a first-order, 2-dimensional Butterworth low-pass filter to the ablation profile. The cutoff frequency of the Butterworth filter was the reciprocal of the smoothing constant. If we simulated the ablation profile for −1 D special myopic LASIK using Munnerlyn algorithm [9] and assumed the optical zone diameter to be 6.5 mm, the smoothing contact could be estimated. The smoothing constant has a unit of length and can be thought of as the radius over which epithelial smoothing occurs. It is determined by the balance between epithelial migration and loss. Detailed explanation of the smoothing constant can be found in a previous article [1]. Ablation simulations were performed using MATLAB software version 5.3 (The Mathworks, Inc., Natick, Massachusetts, USA). The corneal surface was simulated as a sphere of 7.6 mm radius.

Paired *t*-test was used to compare the difference of preoperative and postoperative epithelial thickness. Generalized estimating equation was used to account for the intereye correlation in the variance of *t-*test [10]. Statistical analysis was performed using the Microsoft Excel and SAS 9.1 (SAS Institute Inc., Cary, NC, USA).

## 3. Results

Nineteen eyes (10 right eyes, 9 left eyes) from 11 myopic LASIK patients (6 women, 5 men) were analyzed in the study. The average age was 33.5 ± 6.0 years. The spherical equivalent of LASIK correction ranged from −1.69 D to −6.75 D (mean: −4.39 ± 1.63 D).

**Figure 1:**
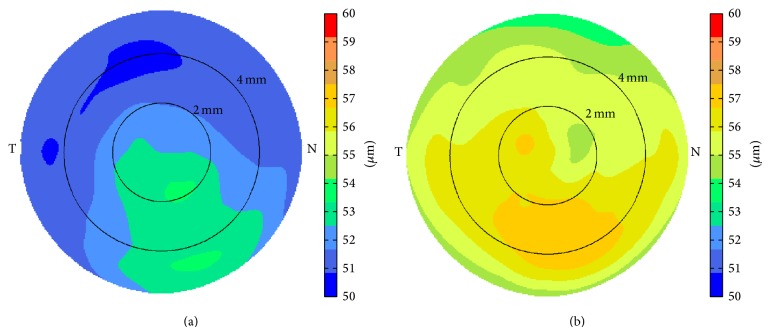
Average epithelial thickness map (a) before LASIK and (b) 3 months after LASIK measured by a Fourier-domain OCT system.

**Figure 2:**
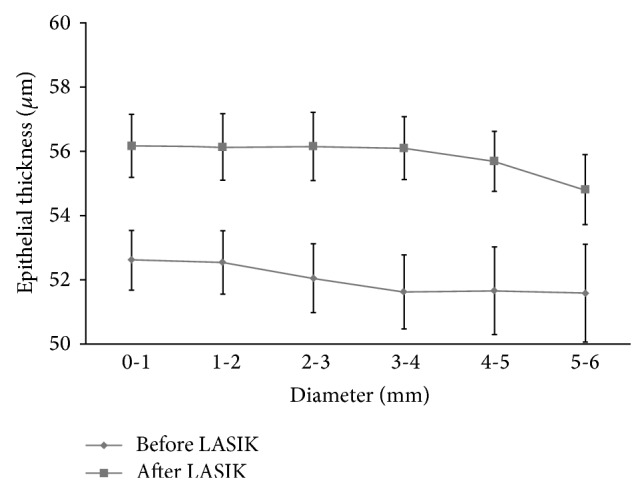
Corneal epithelial thickness before and 3 months after LASIK. Error bar denotes the standard error of the mean, *p* < 0.01 at all diameters.

**Figure 3:**
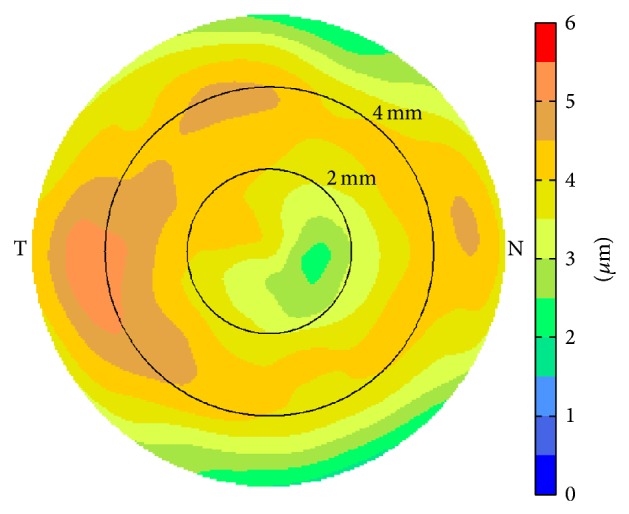
Average epithelial thickness change map after myopic LASIK.

Corneal epithelium was thicker on the inferior side compared to that on the superior side both before (Figure 1(a)) and after LASIK (Figure 1(b)). LASIK-induced epithelial thickening could be observed both centrally and paracentrally. The average central epithelial thickness was measured to be 52.6 ± 4.1 *μ*m (40.9~60.6 *μ*m) before LASIK and 56.2 ± 4.3 *μ*m (50.0~65.5 *μ*m) 3 months after LASIK (*p* = 0.013, Figure 2). The average epithelial thickness at 5~6 mm annular zone was 51.6 ± 6.6 *μ*m (39.6~67.4 *μ*m) before LASIK and 54.8 ± 4.3 *μ*m (49.8~68.0 *μ*m) 3 months after LASIK (*p* = 0.024, Figure 2). The epithelial thickening reached maximum at about 4 mm diameter and tapered off toward the peripheral (Figure 3).

**Figure 4:**
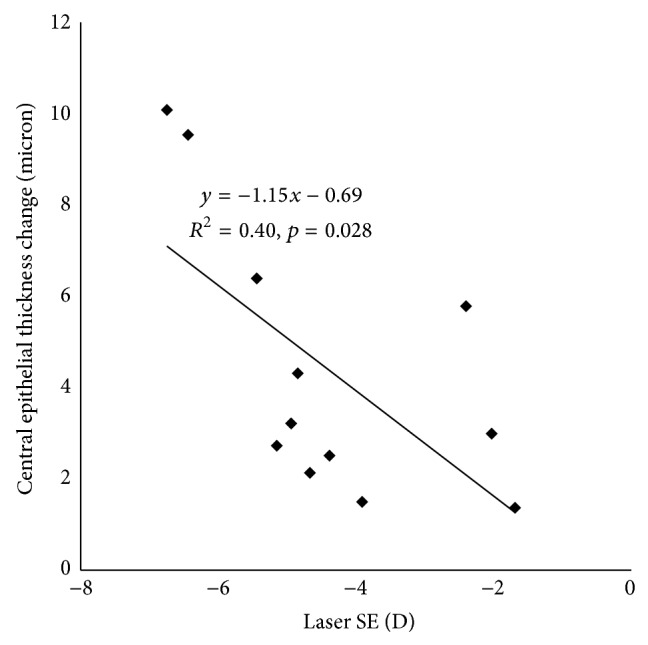
Correlation between central epithelial thickness changes with laser spherical equivalent (SE).

**Figure 5:**
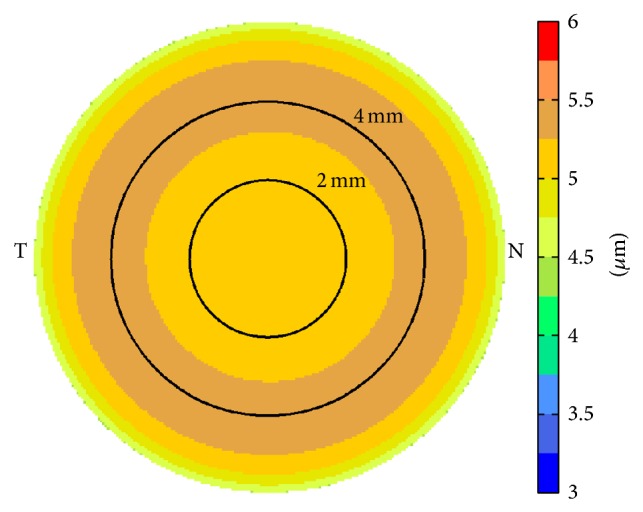
Simulated epithelial thickness change map after myopic LASIK of −4.39 D.

The change in average central epithelial thickness was significantly correlated with LASIK spherical equivalent setting (Figure 4). The slope of −1.15 indicated that, for every diopter of myopic LASIK correction, the central epithelial thickness increased by 1.15 *μ*m, which corresponded to a smoothing constant of about 0.46 mm. Based on this smoothing constant, the simulated epithelial thickness change showed a similar “ring” pattern; that is, the maximum epithelial thickening occurred at an annular area around the center (Figure 5).

## 4. Discussion

Previous studies have demonstrated that it is feasible to use OCT to measure the thickness of different layers of the cornea, such as the epithelium, stroma, and LASIK flap using time-domain OCT [11]. However, the axial resolution of the earlier OCT systems was low and the manual computer-caliper measurement was time consuming. In this study, we used newer Fourier-domain OCT which has faster scan rate and higher axial resolution with automated measurement of epithelial thickness.

The thickness of corneal epithelium was not uniform for normal eyes before LASIK. It was thicker on the inferior compared to that on the superior. The finding agreed with previous OCT studies [8, 12] as well as results with very high-frequency digital ultrasound [4]. The asymmetry might be caused by the movement of upper eyelids during blinking [4, 13]. Similar inferior/superior asymmetry in the thickness of corneal epithelium was also found after LASIK (Figure 1(b)).

After myopic LASIK, epithelial thickening occurred on the central and paracentral area. The average central epithelial thickening was about 3.6 *µ*m at the 3-month followup for a mean spherical equivalent correction of −4.39 D. This finding is in agreement with previous studies that demonstrated the correlation between central epithelial thickening and the amount of myopia correction [5, 14–19]. Using confocal microscopy, Spadea et al. [18] demonstrated that epithelial thickness increased within the first week after LASIK, with a maximum increase of approximately 6.5 *μ*m by the third month for a mean spherical equivalent correction of −10.48 D. Using very high-frequency digital ultrasound [5], Reinstein et al. found that there was a central approximately 5 mm zone of epithelial thickening of up to 7.5 *μ*m 1 year after LASIK for a mean spherical equivalent correction of −3.34 D. In a recent study where a similar OCT system was used, Kanellopoulos and Asimellis [19] found that increases in central (0~2 mm diameter), midperipheral (5 mm diameter), and overall mean epithelial thickness appeared to be in almost a linear correlation with the amount of targeted myopic correction (1.39 *μ*m/D for midperipheral region). The authors hypothesized that epithelial hyperplasia might be caused by a thinned cornea which was biomechanically unstable. This hypothesis was also supported by a study of epithelial thickness changes after collagen cross-linking (CXL) [20]. However, the epithelial thickness changes could actually be explained by the simultaneous topography-guided ablation to reduce the cone. In other words, the epithelial thickness changes could be a response to focal curvature changes [1] in addition to corneal biomechanical properties.

The average central epithelial thickening was significantly correlated with LASIK spherical equivalent setting. For every diopter of spherical myopic LASIK correction, the central epithelial thickness increased by 1.15 *μ*m. The 1.15 *μ*m per diopter epithelial thickening was matched to a smoothing constant of 0.46 mm. This value was larger than our previous estimate (0.32 mm) [1] probably because of the mix of spherical astigmatism subjects in this dataset. Astigmatism ablation pattern had a bigger smoothing constant [1].

Our results showed more epithelial thickening centrally (1 mm diameter zone) than that paracentrally (5-6 mm annular zone). This does not agree with a previous study with very high-frequency digital ultrasound [5] where epithelial thinning was observed between the 5.6 mm and 8 mm diameters except superiorly. We speculated that the discrepancy may be caused by larger optical zone (6.5 mm) and use of transition zone (up to 8.0 mm diameter) in our study compared to the 6 mm optical zone used in the previous study. In Figure 2, if we could measure epithelial thickness beyond 6 mm diameter and the plot could be extrapolated, epithelial thinning would have been observed at the periphery. However, because we did not have access to the proprietary ablation profiles of the laser companies nor had our current OCT system the ability to measure a larger area, we could not provide a more concrete explanation for the disagreement.

On the other hand, both clinical data (Figure 3) and simulation (Figure 5) showed that the maximum epithelial thickening occurred at an annular area about 3~4 mm in diameter, not at the center. The difference was more obvious on the average epithelial change map from the clinical data than the simulation. We speculated the reason being that the actual LASIK ablation pattern might precompensate for the laser-induced spherical aberration, which meant that the actual ablation at the paracentral area would be deeper than Munnerlyn's algorithm used in the simulation which did not account for spherical aberrations. The deeper ablations would introduce more epithelial thickening at these areas. In addition, after the compensatory remodeling of corneal epithelium to surface curvature changes [1], the area with increased epithelial thickening was most likely to be annular because of the ring shape of spherical aberration.

One limitation of this study was that it only included epithelial thickness measurements 3 months after LASIK. However, central epithelial thickening has been reported 1 year [5] up to 7 years [21] after excimer laser ablation, all of which found no statistically significant change in central epithelial thickness after 3 months. Therefore, it may be reasonable to assume that the epithelial thickness is stable 3 months after LASIK. It also should be pointed out that, besides focal corneal curvature, changes in corneal biomechanical properties, such as dry eye [22] and cross-linking [23, 24], may result in corneal remodeling as well. Therefore, it is important to take multiple factors into consideration if they are mixed.

In summary, Fourier-domain OCT was demonstrated to be a valuable tool for noncontact measurements of corneal epithelial thicknesses change caused by LASIK. Corneal epithelial thickened centrally and paracentrally after myopic LASIK. The maximum epithelial thickening occurred at an annular area about 3~4 mm in diameter. The central epithelial thickening and the amount of LASIK correction were statistically correlated. However, wider scans are needed to measure epithelial thickness change toward the edge of the ablation zone.
